# [Corrigendum] IL-21R functions as an oncogenic factor and is regulated by the lncRNA MALAT1/miR-125a-3p axis in gastric cancer

**DOI:** 10.3892/ijo.2025.5780

**Published:** 2025-07-28

**Authors:** Lei Yan, Jing Zhang, Dong Guo, Ji Ma, Shao-Feng Shui, Xin-Wei Han

Int J Oncol 54: 7-16, 2019; DOI: 10.3892/ijo.2018.4612

Following the publication of the above article, an interested reader drew to the authors' attention that their paper was found to contain data with a previous article that had been published in the journal *Oncotarget* (Figs. 2C and 4B), and in a paper that appeared subsequently in the journal *Molecular Cancer.* All cases involved the sharing of Transwell assay data, and the other papers in question were published by the same authors/the same research group.

After having examined their original data, the authors realized that Figs. 2 and 4 had been inadvertently assembled incorrectly in the above paper. Specifically, Figs. 2C and D, and 4B and D, showing the results of Transwell assay experiments indicating the effects of IL-21R knockdown or co-transfection with IL-21R and miR-125a mimic on cell invasion in HGC-27 and MKN-45 cell lines, contained erroneous images. The revised versions of Figs. 2 and 4, featuring replacement data for Figs. 2C and D and 4B and D, showing the correct data obtained for the effects of IL-21R knockdown or co-transfection with IL-21R and miR-125a mimic on cell invasion, are shown on the next page. All authors confirm that the errors made in Figs. 2C and D and 4B and D did not influence the final conclusions reported in the above article, and they thank the Editor of *International Journal of Oncology* for granting them the opportunity to publish a Corrigendum. All the authors agree to the publication of this Corrigendum, and apologize for the inconvenience to the readers.

## Figures and Tables

**Figure 2:**
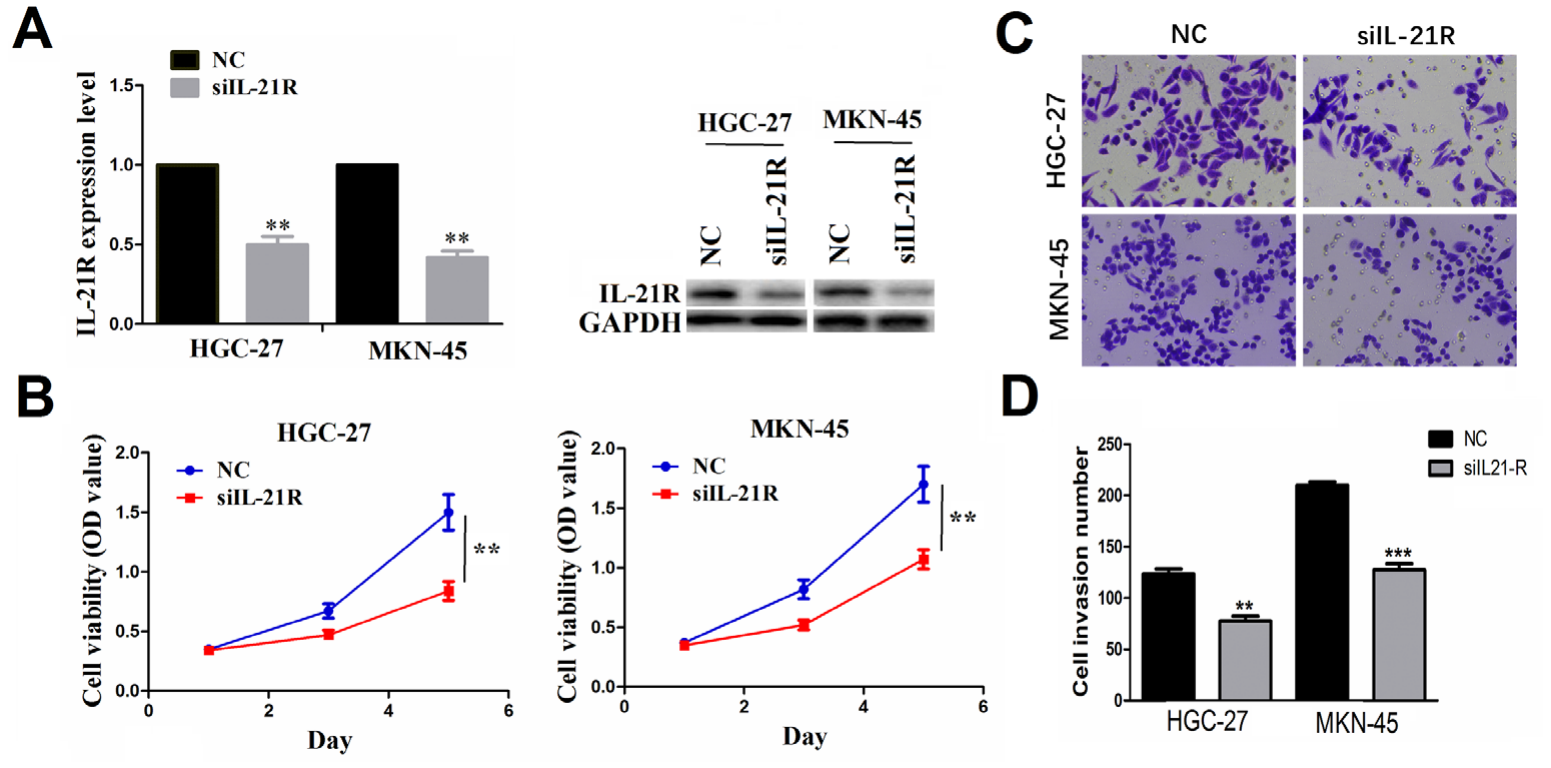
Knockdown of IL-21R suppresses cell proliferation and invasion. (A) RT-qPCR and western blot analysis of the knockdown efficiency following transfection with siIL-21R for 48 h in the HGC-27 and MKN-45 cell lines. (B) CCK-8 assay was used to detect the effects of IL-21R knockdown on cell proliferative activities. (C and D) Transwell invasion assay was conducted to assess the effects of IL-2R knockdown on the invasive potential of gastric cancer (GC) cells. ^**^P<0.01 vs. NC.

**Figure 4:**
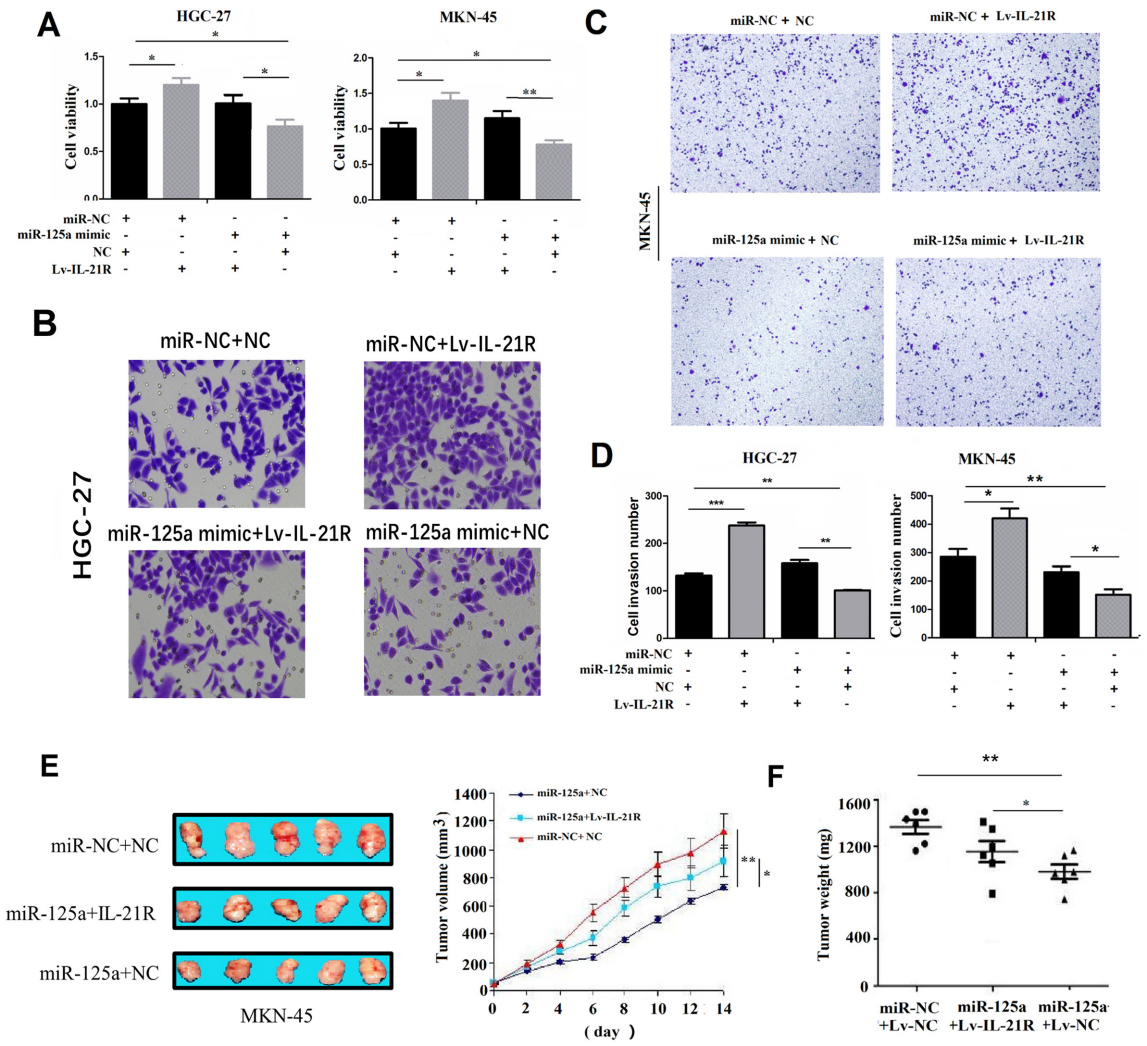
IL-21R overexpression reverses the tumor suppressive effects of miR-34a on gastric cancer (GC) cells. (A) Cell proliferative activities were evaluated by CCK-8 assay in the HGC-27 and MKN-45 cell lines following transfection with Lv-IL-21R and/or miR-125a mimic. (B-D) Cell invasive potential was assessed by Transwell invasion assay in the HGC-27 and MKN-45 cell lines following transfection with Lv-IL-21R and/or miR-125a mimic. (E and F) *In vivo* animal experiments for the validation of the effects of IL-21R overexpression on the antitumor effects of miR-125a. ^*^P<0.05 and ^**^P<0.01..

